# Supplementary material for: Optical Nanomotion Detection to Rapidly Discriminate between Fungicidal and Fungistatic Effects of Antifungals on Single-Cell Candida albicans
Source: Antibiotics (Basel). 2024 Jul 29;13(8):712. doi: 10.3390/antibiotics13080712 (PMC11350713; doi:10.3390/antibiotics13080712)
Supplement: Supplementary file 1 [file antibiotics-13-00712-s001.zip › antibiotics-3105360-supplementary.pdf]

Supplementary Materials

Video S1. Experimental observation of the circular movement of the yeast cell in the microwell during the continuous flow of liquid in the channel at a flow rate of 120 µl/min (movie recording at 20 fps).

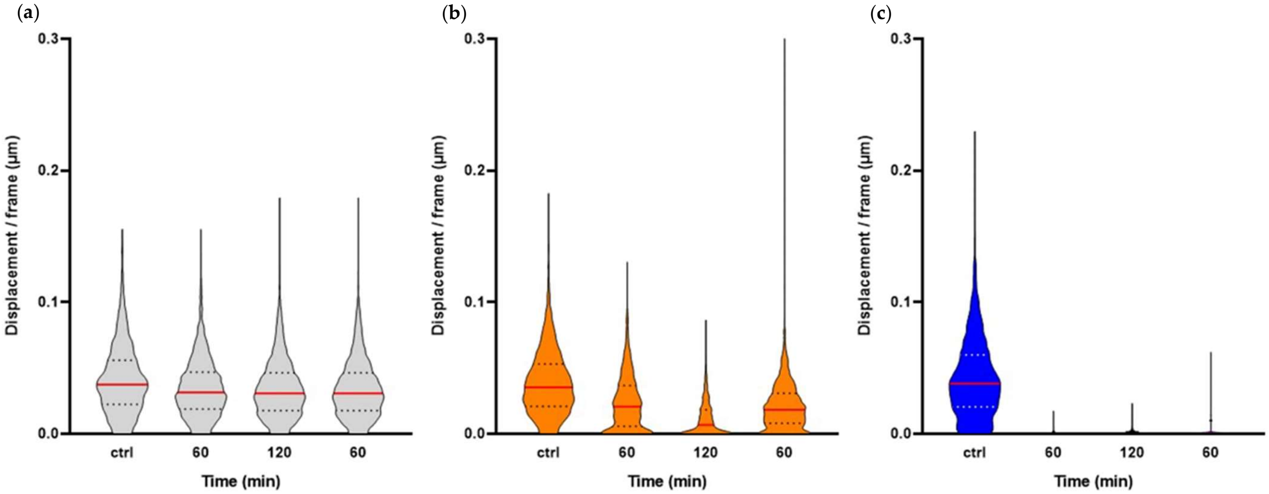

Figure S1. Cellular nanomotion for 20 cells of *C. albicans* CAF2-1. The displacement/frame of 20 cells was followed during (**a**) 2 h treatment with 100µg/ml caspofungin followed with 1 h of YPD treatment (**b**) 2 h treatment with 100 µg/ml fluconazole followed with 1 h of YPD treatment, and (**c**) 2 h treatment with 70% ethanol (v/v) followed with 1 h of YPD treatment.

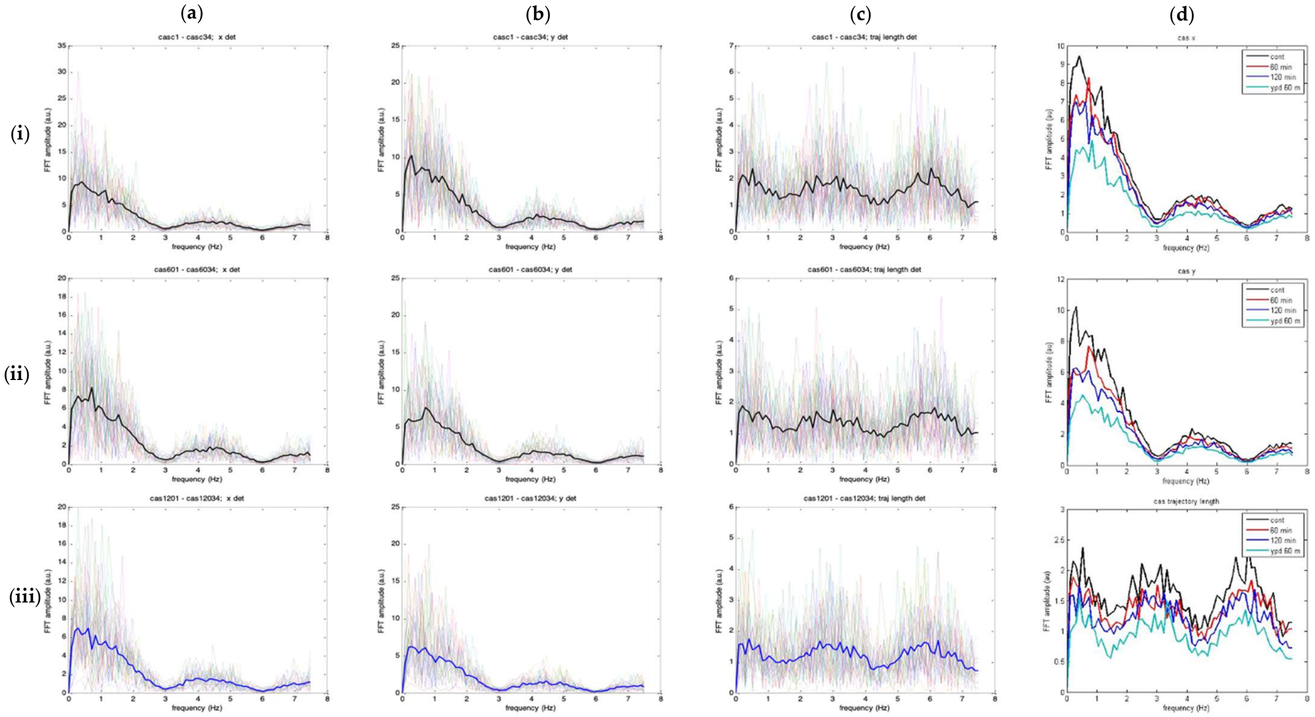

Figure S2. FFT spectra profiles of the nanomovement of 34 *C. albicans* CAF2-1 cells (**i**) before, (**ii**) after 60 min, (**iii**) after 120 min treatment with 100 µg/ml caspofungin. Thin colored lines – individual cells; thick black line – their average. . (**a**) x-axis, (b) y-axis, (c) trajectory length, (d) averaged spectra superimposed.

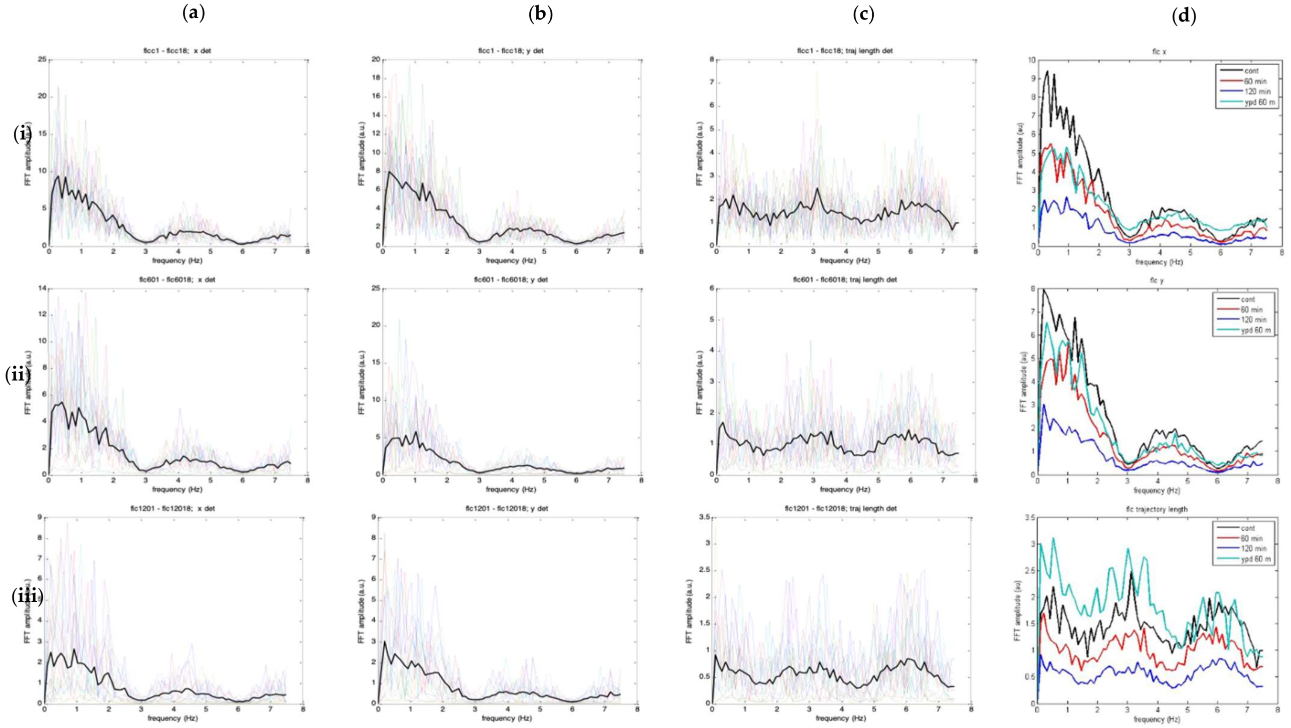

Figure S3. FFT spectra profiles of the nanomovement of 18 *C. albicans* CAF2-1 cells (**i**) before, (**ii**) after 60 min, (**iii**) after 120 min treatment with 100 μg/ml fluconazole. Thin colored lines – individual cells; thick black line – their average. . (**a**) x-axis, (b) y-axis, (c) trajectory length, (d) averaged spectra superimposed.
